# Comparison of Audio Computer Assisted Self-Interview and Face-To-Face Interview Methods in Eliciting HIV-Related Risks among Men Who Have Sex with Men and Men Who Inject Drugs in Nigeria

**DOI:** 10.1371/journal.pone.0081981

**Published:** 2014-01-08

**Authors:** Sylvia Adebajo, Otibho Obianwu, George Eluwa, Lung Vu, Ayo Oginni, Waimar Tun, Meredith Sheehy, Babatunde Ahonsi, Adebobola Bashorun, Omokhudu Idogho, Andrew Karlyn

**Affiliations:** 1 Population Council, Abuja, Nigeria; 2 Population Services International (PSI), Washington, DC, United States of America; 3 Population Council, Washington, DC, United States of America; 4 Population Council, New York, New York, United States of America; 5 Federal Ministry of Health, Abuja, Nigeria; 6 Society for Family Health, Abuja, Nigeria; 7 United States Agency for International Development (USAID), Washington, DC, United States of America; University of Missouri-Kansas City, United States of America

## Abstract

**Introduction:**

Face-to-face (FTF) interviews are the most frequently used means of obtaining information on sexual and drug injecting behaviours from men who have sex with men (MSM) and men who inject drugs (MWID). However, accurate information on these behaviours may be difficult to elicit because of sociocultural hostility towards these populations and the criminalization associated with these behaviours. Audio computer assisted self-interview (ACASI) is an interviewing technique that may mitigate social desirability bias in this context.

**Methods:**

This study evaluated differences in the reporting of HIV-related risky behaviours by MSM and MWID using ACASI and FTF interviews. Between August and September 2010, 712 MSM and 328 MWID in Nigeria were randomized to either ACASI or FTF interview for completion of a behavioural survey that included questions on sensitive sexual and injecting risk behaviours. Data were analyzed separately for MSM and MWID. Logistic regression was run for each behaviour as a dependent variable to determine differences in reporting methods.

**Results:**

MSM interviewed via ACASI reported significantly higher risky behaviours with both women (multiple female sexual partners 51% vs. 43%, p = 0.04; had unprotected anal sex with women 72% vs. 57%, p = 0.05) and men (multiple male sex partners 70% vs. 54%, p≤0.001) than through FTF. Additionally, they were more likely to self-identify as homosexual (AOR: 3.3, 95%CI:2.4–4.6) and report drug use in the past 12 months (AOR:40.0, 95%CI: 9.6–166.0). MWID interviewed with ACASI were more likely to report needle sharing (AOR:3.3, 95%CI:1.2–8.9) and re-use (AOR:2.2, 95%CI:1.2–3.9) in the past month and prior HIV testing (AOR:1.6, 95%CI 1.02–2.5).

**Conclusion:**

The feasibility of using ACASI in studies and clinics targeting key populations in Nigeria must be explored to increase the likelihood of obtaining more accurate data on high risk behaviours to inform improved risk reduction strategies that reduce HIV transmission.

## Introduction

There is growing evidence that men who have sex with men (MSM) and men who inject drugs (MWID) in Nigeria are hyper-vulnerable to HIV infection [1]–[7] because of high levels of political, religious and cultural hostility as well as the criminalization of their behavior [4], [8], [9]. According to the two rounds of the Integrated Biological Behavioural Surveillance Survey (IBBSS) conducted in Nigeria in 2007 and 2010, the estimated HIV prevalence among MSM increased from 13.5% in 2007 to 17.2% in 2010 and among PWID, HIV prevalence decreased from 5.2% in 2007 to 4.2% in 2010 [1], [2]. The surveys also revealed low self-perceived risk, significant levels of risky sexual and injecting practices and poor health-seeking behaviours among MSM and MWID in Nigeria [1], [2].

Unbiased measurements of socially sensitive behaviours are necessary to accurately study sensitive behaviours that may determine acquisition and transmission of sexually transmitted infections (STIs) including HIV [10], [11]. In some populations, an audio computer-based technology that enables respondents to self-administer questionnaires in complete privacy, such as the audio computer assisted self-interview (ACASI), has succeeded in eliciting unbiased responses for socially sensitive behaviours [10], [12], [13]. Studies comparing responses from clinician interviews and ACASI of self-reports of socially sensitive behaviours revealed that ACASI responses were more complete for socially sensitive behaviours like admitting to having same-gender sex partners and illicit drug use, group sex, rape, commercial sex than face-to-face (FTF) interviews [14]–[18]. Advantages of ACASI formatted surveys include consistency in the way questions are asked thus, maximizing standardization; limiting handling of data forms, protecting participant confidentiality and direct data capturing thereby, decreasing staff effort and enhancing data quality [10], [12], [16], [19], [20].

The ACASI technology is ideal for research with key populations such as MSM and people who inject drugs (PWID) for reliable and frank reporting of sensitive behaviours. However there is limited testing of the instrument in low resource settings such as Nigeria [12], [13], [15], [21]–[23]. We evaluated differences in the reporting of risky HIV-related behaviours among MSM and MWID using ACASI and FTF. The hypothesis was that MSM and MWID interviewed by ACASI method would be more likely to report sensitive HIV-risk behaviours compared to those interviewed FTF.

## Methods

### Study sites

This study was conducted at three Men's Health Network, Nigeria (MHNN) clinics in Abuja, located in north central Nigeria, and Lagos and Ibadan, both located in southwest Nigeria. MHNN provides HIV prevention services including behaviour change communications, HIV counselling and testing (HCT), syndromic management of STIs, and condom and lubricant distribution to key male populations (MSM and MWID) and their male and female sex partners.

### Study populations and sampling strategy

MSM were defined as men aged 18 years and above who reported sexual activity (oral or anal) with another man at least once in the 12 months preceding the survey. MWID were defined as men aged 18 years and older who injected drugs recreationally at least once in the last 12 months. Participants were recruited through respondent-driven sampling (RDS), an adaptation of snowball or chain referral sampling typically used to recruit hard-to-reach populations where peers recruit their peers into the study [24], [25]. Recruitment of MSM and MWID spanned six weeks from August to September, 2010.

### Ethics statement

Due to the sensitive nature of the study, special precautions were taken in conducting the study to maximize the safety and confidentiality of participants. Ethical approval was obtained from the Institutional Review Boards of the Nigerian Institute for Medical Research, Nigeria and the Population Council, New York. Participation in the study was voluntary and did not in any way compromise participants' access to services offered by the Men's Health Network, Nigeria (MHNN). Written informed consent was obtained from all participants prior to conducting all study procedures. Participants were able to receive laboratory tests for STIs (syphilis, gonorrhoea, chlamydia, HBV, and HCV) free-of-charge if they desired. They were also compensated NGN500 for participating in the first visit of this study and an additional NGN500 at their follow-up visit for each additional eligible peer they successfully recruited into the study. The total amount of compensation was between NGN 1,000 (USD 6.60) and NGN 2,000 (USD 13.30) depending on the number of peers each participant was able to recruit.

### Data collection

Behavioural data were collected by the administration of face to face (FTF) interviews and by ACASI. The questionnaire was available in both English and Pidgin English in both interview modes. Participants were randomized to either the ACASI or FTF interview arm for completion of the behavioural survey. Randomization was determined by a set of random numbers generated from Random Allocation Software [26]. ACASI interviews were conducted in private cubicles with the use of laptops and headphones. Each respondent was given a short orientation to familiarize them with the system and to ensure respondent's comfort with the laptop, mouse, and the format of the questionnaire. Survey questions for both arms of the study were identical, and participants had the option of not answering any question. At the end of the ACASI interview, a random 10% of participants were selected to answer a short exit survey assessing participants' experience using ACASI and their opinions about future use of ACASI. The survey lasted for about 30 minutes in the FTF method and 40 minutes in the ACASI method.

### Variables of Interest

To test the study hypothesis, a set of sensitive sexual and injecting risk behaviours was selected based on literature review. For MSM, the following indicators were selected: 1) sexual identity; 2) having multiple sex partners in the past two months; 3) having anal sex with women in the past two months; 4) having unprotected anal sex with men and women at last sex; 5) injecting drugs; 6) having STI symptoms in the past 12 months; 7) using drugs in the past 12 months; and 8) ever testing for HIV. For MWID, the following questions were selected: 1) having multiple sex partners; 2) having unprotected sex at last sex; 3) having casual sex partners, including commercial sex in past two months; 4) age at first injecting drugs; 5) years of injecting drug; 6) sharing of needles and syringes; 7) STI symptoms in the past 12 months; and 8) ever testing for HIV. To elicit STI symptoms, respondents were asked if they had experienced any pain, itching, ulcer/sore or discharge from the penis or anal region in the past 12 months.

### Statistical Analyses

Data were analyzed separately for MSM and MWID using STATA software version 11. MSM data were pooled across the 3 sites to provide sufficient statistical power. To determine whether the randomization was successful, demographic characteristics including age (age = 0.41), education (p = 0.26) and employment status (p = 0.07) were compared between the two groups. Descriptive analysis was conducted to describe the sample characteristics. For bivariate analysis we used chi-squared test to determine differences in reporting for categorical variables and t-test to determine differences in reporting for continuous variables by interview mode. Logistic regression model was run for each sensitive behaviour as a dependent variable to determine if ACASI elicited significantly different responses from FTF while controlling for age, education, HIV status, and study sites. The influence of age and socio-economic status (using education and study sites as proxies at the individual and structural levels respectively) on reporting of health outcomes is well documented [27], [28]. Odds ratios, confidence intervals and p-values are reported. The level of significance was determined at p<0.05.

## Results

### Characteristics of the study population

For recruitment of MSM in Abuja, five of seven seeds actively recruited with a maximum of eleven waves of recruitment and an average of six waves for each active seed. In Ibadan, three of four seeds actively recruited with a maximum of eight recruitment waves and an average of seven waves while in Lagos all three seeds were active with a maximum of 14 waves of recruitment and an average of six waves.

A total of 712 MSM and 328 MWID were recruited. Median age was 23 years and 40 years, respectively (Table 1). A large proportion of MSM (65%) and MWID (45%) had completed at least secondary level education and about a third of both MSM and MWID were unemployed. Among the MSM, 42% self-identified as homosexual and 87% had had anal sex with a man in the past twelve months.

**Table 1:** Demographic characteristics of the study population.

Variables	MSM (n = 712) % (n)	MWID (n = 328) % (n)
**Age**		
(median & range)	23 (18–52)	40 (18–50)
**Education**		
Primary or less	6.5 (46)	26.8 (88)
Secondary	64.9 (462)	44.8 (147)
Tertiary	28.7 (204)	28.4 (93)
**Unemployment**	33.4 (238)	34.5 (113)
**Being HIV positive**	22.6 (150)	1.8 (6)
**Self-reported homosexual identity**	42.0 (275)	n/a
**Had anal sex with a man in the past 2 months**	86.7 (617)	n/a
**Injected drug in the past month**	7.7 (55)	75.7 (237)

### Reporting of sensitive information by interview mode

#### Men who have sex with men

Table 2 compares the reports of sexual behaviours by participants using ACASI and FTF interviewing techniques. Interestingly, a significantly higher proportion of respondents in the ACASI group compared with the FTF group reported being married or cohabiting with a woman (15% vs. 11%; p≤0.0001) or cohabiting with a man (26% vs. 15%; p≤0.0001) (data not shown). Similarly, a higher proportion of respondents in the ACASI group reported having more than one female sex partner in the past two months compared to those in the FTF group (51% vs. 43%; p = 0.04). In the ACASI group, a significantly higher proportion of respondents reported anal sex with women in the past two months prior to the survey (72% vs. 21%; p≤0.001), unprotected anal sex with women (72% vs. 57%; p = 0.05), being paid for sex in the past six months (54% vs. 46%; p = 0.05), having two or more male sex partners in the past two months compared to FTF (70% vs. 54%; p≤0.001), and reporting drug use in the last 12 months (16% vs. 1%; p<0.001) compared to those in the FTF group. The use of cocaine (5% vs 1%; p≤0.001), heroine (2% vs 1%; p≤0.001) and marijuana (21% vs. 12%; p≤0.001) in the past 12 months was higher among those interviewed via ACASI compared to FTF. However, there was no difference between the ACASI and FTF groups for unprotected anal sex with a male partner at last sexual intercourse (42% vs. 45%; p>0.05) and self-report of ever testing for HIV (58% vs. 52%; p = 0.12).

**Table 2:** Multivariate analysis for reported HIV risks by interview modes among MSM.

Variables	FTF (N = 372) % (n)	ACASI (N = 342) % (n)	p-value	AOR (95% CI)
Self-reported homosexual identity	29.1 (103)	57.1 (172)	*≤0.001*	3.3 (2.4–4.6)***
Had multiple female sex partner in the past two month	43.0 (159)	50.6 (173)	*0.04*	1.4 (1.1–1.9)*
Had multiple male sex partner past two months	54.1 (200)	70.0 (239)	*≤0.001*	2.1 (1.5–2.8)***
Had anal sex with women in past two month	21.3 (60)	71.6 (121)	*≤0.001*	13.1 (7.9–21.7)***
Had unprotected anal sex with men at last sex	45.1 (167)	41.5 (142)	*0.33*	0.9 (0.7–1.2)
Had unprotected anal sex with women at last sex	57.1 (36)	71.7 (86)	*0.05*	2.1 (1.1–4.1)*
Had STI symptoms in the past 12 months	20.5 (76)	42.7 (146)	*≤0.00*	2.9 (2.1–4.1)***
Ever tested for HIV	52.2 (191)	57.9 (198)	*0.12*	1.2 (0.8–1.6)
Injected drug past 12 months	0.6 (2)	16.0 (53)	*0.00*	40.0 (9.6–166.0)***
Used cocaine in past 12 months	1.4 (5%)	4.7 (16)	*≤0.001*	
Used heroine in past 12 months	0.8 (3)	2.3 (8)	*≤0.001*	
Used marijuana in past 12 months	11.6 (43)	21.4 (73)	*≤0.001*	

***Note:*** AOR = Adjusted odds ratio, CI = confidence interval,

: significant at *p*<.05,

: significant at *p*<.01,

: significant at *p*<.001.

Age, education, HIV status, and study sites were adjusted for in the regression models looking at likelihood of reporting HIV risks by interview method (reference FTF).

#### Men who inject drugs

Among MWID, the median age of first injection was lower among those interviewed via ACASI compared to FTF (28 years vs. 30 years; p = <0.01). For injecting risk behaviours, there was higher reporting of sharing (13% vs. 5%; p<0.05) and reusing needles by the same individual (40% vs. 27%; p = 0.03) among those interviewed via ACASI than FTF. Although, a higher proportion of MWID in the FTF reported being married or cohabiting with a woman compared with the ACASI (47% vs. 33%; p = 0.02) (data not shown), however, there were no significant differences between the ACASI and FTF groups in their reports of the number of female sex partners (18% vs. 17%; p = 0.9), unprotected sex at last sex (18% vs. 15%; p = 0.6) and injecting drugs in the past month (76% vs. 76%; p = 0.9).

### Likelihood of reporting sensitive behaviours

Table 2 reports the results of the multivariate analysis of the two interview modes. MSM interviewed by ACASI were more likely to self-identity as homosexual (AOR:3.3, 95% CI:2.4–4.6), to report multiple female partners (AOR:1.4, 95% CI:1.1–1.9); multiple male partners (AOR: 2.1, 95% CI: 1.5–2.8); anal sex with women (AOR:13.1, 95% CI: 7.9–21.7); and unprotected anal sex with women (AOR:2.1, 95% CI:1.1–4.1). Additionally, MSM in the ACASI arm were more likely to report STI symptoms in the last 12 months (AOR: 2.9, 95% CI: 2.1–4.1) and to use drugs in the past 12 months (AOR: 40.0, 95% CI: 9.6–166.0).

Among MWID, those interviewed by ACASI were more likely to report needle sharing in the past month (AOR:3.3, 95% CI: 1.2–8.9), reusing of their own needles (AOR:2.2, 95% CI:1.2–3.9), and ever testing for HIV (AOR:1.6, 95% CI:1.02–2.5) (Table 3).

**Table 3:** Reported HIV risks by interview modes among MWID (N = 328).

Variables	FTT (N = 166) % (n)	ACASI (N = 162) % (n)	χ2 or t (p-value)	OR (95% CI)
Had multiple female sex partners in past 2 months	17.5 (29)	16.7 (27)	*0.04 (0.85)*	1.0 (0.5–1.7)
Had unprotected sex at last sex	17.5 (29)	15.4 (25)	*0.2 (0.61)*	0.9 (0.5–1.6)
Had casual sex partners in past 2 months	7.9 (5)	26.4 (14)	*7.2 (0.03)*	**3.7 (1.2–11.4)** *
Age first injected drugs [mean (std.)]^a^	30.1 (0.5)	27.6 (0.7)	*3.0 (0.003)*	**0.96 (0.93–0.99)** *
Number of years injecting drugs [mean (std.)]^a^	8.4 (0.5)	7.9 (0.6)	*0.7 (0.49)*	1.0 (0.95–1.02)
Injected drugs in the past month	75.8 (122)	75.7 (115)	*0.00 (0.98)*	1.0 (0.6–1.7)
Shared needle/syringes in the past month	4.9 (6)	13.3 (15)	*5.1 (0.02)*	**3.3 (1.2–8.9)** *
Used your own needle and syringes over again	26.6 (91)	40.0 (46)	*4.8 (0.03)*	**2.2 (1.2–3.9)** **
Had STI symptoms in the past 12 months	4.8 (8)	11.1 (18)	*4.4 (0.04)*	**2.4 (1.02–5.8)** *
Ever tested for HIV	47.2 (76)	36.0 (58)	*4.1 (0.04)*	**1.6 (1.02–2.5)** *

***Note:*** AOR = adjusted odds ratio, CI = confidence interval,

: significant at *p*<.05,

: significant at *p*<.01.

Age, education, and HIV status were adjusted for in the regression models looking at reported HIV risks among ACASI method vs. FTF interview method.

^a^ significant levels were determined using t-test comparing 2 continuous variables.

### Acceptability of ACASI

About 76% of respondents reported that they were interviewed via FTF in a behavioural survey at least once in the past. Nonetheless, more than 80% (Table 4) felt comfortable using the ACASI interview method and found it not difficult to use. On a scale of 1–5 (with 1 representing do not like the ACASI at all and 5 liking it very much), over two-thirds of respondents liked the ACASI method very much. Almost half of the respondents (47%) liked the ACASI because of privacy and 31% liked it because it was clearer (by being able to both read and listen to the questions). Nearly all respondents (97%) would like to use ACASI in the future if given a choice. On average, the ACASI method took about 10 minutes longer than the FTF method (40 minutes vs. 30 minutes).

**Table 4:** Experience and preference regarding the use of ACASI (N = 94).

	*% (n)*
**How often you use computer?**	
Everyday/almost everyday	42.5 (40)
Once/twice a week	23.4 (22)
Once/twice a month	19.2 (18)
Almost never	14.9 (14)
**How comfortable were you using the ACASI?**	
Very comfortable	80.9 (76)
Comfortable	18.1 (17)
Uncomfortable	1.1 (1)
**How difficult in using the ACASI?**	
Very difficult	3.2 (3)
Somewhat difficult	12.8 (12)
Not at all	84.0 (79)
**How did you rate the ACASI?** *	
1	3.2 (3)
2	2.1 (2)
3	12.8 (12)
4	13.8 (13)
5	68.1 (64)
**What did you like most about using computer?**	
More fun	17.0 (16)
More private	46.8 (44)
Clearer because I can read and listen to the questions	30.9 (29)
Nothing	5.3 (5)
**Have you done a FTF interview before?**	
Yes	75.5 (71)
No	24.5 (23)
**Which type of interview did you like?**	
ACASI	64.5 (49)
FTF	9.2 (7)
Both equally	26.3 (20)
**Would like to use computer in the future to answer questions**	
Yes	96.8 (90)
No	3.2 (3)

**Note:** *1 = did not like it at all, 5 = liked it very much.

## Discussion

This is the first study to evaluate self-reports of sexual risk behaviours among MSM and MWID in Nigeria using ACASI and FTF. We observed some important findings. First, MSM interviewed via ACASI were more likely to self-identify as homosexual or gay and report significantly higher levels of engagement in sexual risk behaviours with both women and men for the following indicators: multiple male and female sexual partnerships and unprotected anal sex with women. Second, MSM respondents in the ACASI group reported significantly higher use of psychoactive drugs, highlighting drug use among MSM. Third, MWID were more likely to report sharing needles and reporting younger age at injection debut via ACASI than FTF. Fourth, the study showed high levels of acceptability and preference for ACASI among respondents because of privacy and ease of use. These findings have important implications for HIV research and programming in Nigeria. Furthermore, the higher levels of bisexuality and risky sexual behaviors reported by ACASI respondents in this study and elsewhere [6], [7], [29], [30] highlights the urgent need for MSM interventions in Nigeria to incorporate information on safer sex with both male and female sex partners.

The study also identified significantly higher reporting of gay or homosexual identity, cohabiting with a male partner and engaging in HIV-related risks with ACASI. This highlights the challenges that researchers may face in obtaining accurate estimates of HIV-related risks with standard behavioural surveys administered via FTF, including the Integrated Biological and Behavioral Surveillance Survey (IBBSS). Compared to the IBBSS, more MSM respondents interviewed via ACASI reported having experienced STI symptoms in the past 12 months (43% vs. 15%), had two or more male partners (70% vs. 50%) and did not use condom at last sex with men (67% vs. 50%) and female sex workers (72% vs. 35%). In addition, health care providers serving key populations may also encounter challenges in identifying risky behaviours to guide adequate counselling and the development of appropriate risk reduction plans. Another significant finding of this study was the higher reporting of drug use among MSM. MSM interviewed with the ACASI were forty times more likely to report drug use than those interviewed with FTF. This indicates that drug use among MSM may be much higher than is often reported. In addition, because drug use is associated with higher sexual-risk taking, deeper knowledge of this risk factor among MSM is required to better inform the design of effective comprehensive interventions.

The acceptability and preference of ACASI by MSM and MWID is high, indicating the feasibility of ACASI use in future surveys. Privacy and ease of use are possible factors that contribute to the higher reporting of risk behaviours among both MSM and MWID. Qualitative studies in Zimbabwe [31] and in the United States [14] reported that the perceived privacy and confidentiality of ACASI are reasons behind more accurate reporting of sensitive behaviours. The acceptability of ACASI has also been found to be high even among respondents with low levels of computer literacy in resource limited settings [31].

The findings of this study demonstrate that efforts and resources must be geared towards using ACASI in future surveys to elicit more accurate behavioural information to guide evidence-based HIV prevention programming. Furthermore, obtaining accurate estimates of HIV-related risks is important for measuring the effectiveness of interventions and modelling optimal packages for HIV preventions.

### Limitations

This study has some limitations. We did not assess the reporting of HIV-related risk behaviour using both methods on the same individuals hence, we were unable to assess the validity and consistency of each interview method. However, given that the assessment was part of a larger cross-sectional survey that assessed HIV and STI prevalence and risks, randomization of respondents into two study arms was the most feasible option.

## Conclusion

This is the first study to compare self-reports of HIV risk behaviours among MSM and MWID in Nigeria using ACASI and FTF interview modes. The significantly higher reporting of risk behaviours of ACASI respondents suggests that risks of MSM and MWID may be underestimated in traditional FTF surveys. As accurate reporting of HIV-risk behaviours is important for HIV programming, research and allocation of resources, ACASI or CASI is highly recommended in both clinical and research settings to reduce social desirability bias. This may be of particular importance in surveys among key populations who engage in behaviours that are stigmatized and often illegal.
